# Relationship Between Handgrip Strength and Lung Function in Adults: The Role of Sex and Age

**DOI:** 10.7759/cureus.83300

**Published:** 2025-05-01

**Authors:** Shiqi Deng, Urme Binte Sayeed, Yukiko Wagatsuma

**Affiliations:** 1 Department of Clinical Trial and Clinical Epidemiology, Graduate School of Comprehensive Human Sciences, University of Tsukuba, Tsukuba, JPN; 2 Department of Clinical Trial and Clinical Epidemiology, Institute of Medicine, University of Tsukuba, Tsukuba, JPN

**Keywords:** handgrip strength, health checkup, healthy population, lower limit of normal, lung function

## Abstract

Background

The association between handgrip strength and lung function has been explored in previous studies, yet few studies have addressed how this relationship varies by sex and age in healthy populations. Additionally, few studies have investigated the utility of the lower limit of normal (LLN) for identifying abnormal lung function in relation to handgrip strength. This study aimed to evaluate sex- and age-specific associations between handgrip strength and lung function in a healthy adult population.

Methods

This cross-sectional study included individuals undergoing health check-ups at a regional hospital in Japan from April 2018 to March 2020. Data on demographics, anthropometrics, handgrip strength, spirometry, and lifestyle factors were collected. Handgrip strength was assessed using a digital dynamometer, with two measurements taken for each hand while keeping the arms straight down at the sides without touching the body. The highest recorded value from each hand was averaged as the final measure. Pearson’s correlation and multiple regression analyses were conducted to explore the relationship between handgrip strength and lung function parameters. Logistic regression analyses were used to estimate odds ratios (OR) and 95% confidence intervals (CI) for abnormal lung function per 5 kg increase in handgrip strength.

Results

A total of 1622 individuals were included in the study. The correlation coefficients between handgrip strength and forced expiratory volume in 1 second (FEV1) and forced vital capacity (FVC) were approximately 0.3-0.45 (p < 0.01) after stratification by sex. Linear regression analysis showed a positive association between handgrip strength and FEV1, percentage of the predicted FEV1 value (FEV1% predicted), FVC, and percentage of the predicted FVC value (FVC% predicted) across all age groups in women (p < 0.05). In men, these associations were significant in the 25-44 and 45-59 year age groups (p < 0.05). Logistic regression indicated that a 5 kg increase in handgrip strength was significantly associated with reduced abnormal lung function in both men (OR = 0.790, 95% CI = 0.673-0.927; p < 0.01) and women (OR = 0.598, 95% CI = 0.461-0.775; p < 0.01); however, the significance was nullified in men over 60 years of age after further age stratification.

Conclusion

Handgrip strength is positively associated with FEV1 and FVC, both in absolute and predicted percentage values, across all age groups in women and in men under 60 years of age. A 5 kg increase in handgrip strength is significantly associated with lower abnormal lung function in women. Handgrip strength measurements integrated into regular health checkups may help in the early detection of lung function abnormalities, even in early adulthood. Further longitudinal studies are warranted to validate these findings.

## Introduction

Lung function declines with age; however, the condition is not limited to the elderly [1]. Respiratory diseases, such as chronic obstructive pulmonary disease (COPD), are among the leading causes of death, as well as years lived with disability, worldwide [2]. An accelerated decline in lung function can have important clinical consequences, resulting in a substantial burden on society. According to the World Health Organization (WHO), 3.23 million people died of COPD worldwide in 2019 [3]. In Japan, approximately 10% of the population suffers from COPD, and its prevalence is increasing each year [4]. Therefore, the early identification of populations at high risk of lung function decline is important from a public health perspective.

Handgrip strength is a practical and cost-effective measure that reflects an individual's overall muscle strength and serves as a potential marker of general health outcomes [5]. Low handgrip strength has been demonstrated to be associated with various comorbidities, such as hypertension, coronary artery occlusive disease, stroke, and all-cause mortality [6]. Owing to the simplicity and reliability of handgrip strength measurements, their efficacy in the assessment of lung function status has attracted increasing interest.

Several previous studies have reported a significant positive association between handgrip strength and lung function, including forced expiratory volume in 1 second (FEV1) and forced vital capacity (FVC) [7-9]; additionally, handgrip strength has been shown to reflect pulmonary diseases [10]. However, few studies have examined the differences in this relationship across sex and age groups in healthy populations. Men and women differ physiologically in terms of muscle strength and lung function [11,12]. Additionally, lung function and handgrip strength follow distinct age-related trajectories [9,13]. Given these differences, the relationship between handgrip strength and lung function may vary by sex and age. Moreover, studies have classified lower lung function using either population-based percentile cutoffs or a fixed FEV1 as the percentage of FVC (FEV1/FVC) to identify lower lung function [14,15]. Using a fixed ratio is more convenient in clinical practice; however, since the FEV1/FVC naturally decreases with age, this approach may lead to overdiagnosis in elderly individuals [16,17]. Furthermore, a lack of early interventions may lead to severe respiratory morbidity and death [18]. To date, studies on the use of the lower limit of normal (LLN) to identify abnormal lung function and its relationship with handgrip strength are underexplored.

This study aimed to examine sex- and age-stratified associations between handgrip strength and lung function in healthy adults undergoing health check-ups. The hypotheses were that the relationship between lung function and handgrip strength varies by sex and age and that lower handgrip strength is significantly associated with abnormal lung function.

## Materials and methods

Study subjects and design

This study recruited individuals who underwent health check-ups at a regional hospital located in Mito City, Ibaraki Prefecture, Japan, and at several outreach sites around the region between April 2018 and March 2020. The participants were invited to participate in the study during their health check-ups. This cross-sectional study is a part of an ongoing cohort study designed to investigate various health parameters and their associations, with the goal of improving the overall health status of the community. In addition to health check-up data, demographic, anthropometric, handgrip strength, spirometry, and lifestyle data were collected during the health check-ups. Participants were considered healthy if they attended health check-ups and had no severe medical conditions.

Measurements

During health check-ups, height and weight were measured using a Tanita DC-250 body composition analyzer (Tanita Co., Japan). Body mass index (BMI) was calculated by dividing weight in kilograms by the square of height in meters. Systolic and diastolic blood pressure (SBP and DBP, respectively) were measured by medical staff using an automatic sphygmomanometer (MPV-3301, Nihon Kohden Corporation, Tokyo, Japan) after the participants sat and rested.

Handgrip strength

Handgrip strength was measured using a Smedley digital handgrip test machine (Takei Corporation, Japan) equipped with a hand dynamometer. All measurements were performed by trained personnel following a standardized protocol to ensure consistency. The dynamometer was calibrated before each session, and its settings were adjusted to optimize participants' force application. Handgrip strength was measured twice on the left and right sides, with both arms straight down to the side of the body and in a posture in which the handgrip strength meter did not touch the body. The participants were instructed to grip the dynamometer firmly and apply maximum strength for three seconds. The average maximum value for each hand was used as an indicator of handgrip strength.

Spirometry

FEV1, FVC, and FEV1/FVC were measured using a multifunctional spirometer (FDAC-7D; Fukuda Denshi, Japan) while the participants were in a seated position. Medical staff conducted the tests following a standardized protocol to ensure consistency. Before test initiation, the participants were informed of the procedure and instructed on the proper technique.

Questionnaire

As specified by the Ministry of Health, Labour, and Welfare of Japan, a self-administered questionnaire was used to collect information regarding the following factors used in the analyses: smoking status; exercise habits; alcohol intake; history of heart disease, stroke, and chronic renal failure; and the use of antihypertensive, antidiabetic, and anticholestatic agents. The questionnaire is presented in Appendix A.

Definitions

The LLN was used to define abnormal lung function. This method allows comparison of lung function across a wide range of ages and provides sex, age, and height-specific values that are below the levels obtained in the healthy reference population at the lowest 5% of the frequency distribution. In this study, FEV1, FVC, and FEV1/FVC below the LLN were classified as abnormal lung function.

FEV1% predicted, FVC% predicted, and FEV1/FVC% predicted were calculated as follows:

$\frac{\text{FEV1; FVC; FEV1/FVC}}{\text{predicted } (\text{FEV1; FVC; FEV1/FVC})} \times 100$

The predicted values and LLN were calculated using the following reference equations suggested by the Japanese Respiratory Society [19].

Hypertension was defined as an SBP ≥ 140 mmHg and/or a DBP ≥ 90 mmHg and a prescription of medications for hypertension [20].

BMI (kg/m^2^) was categorized as < 18.5 (underweight), 18.5-24.9 (normal), and ≥ 25.0 (overweight).

Statistical analyses

The participants were categorized into three age groups (25-44, 45-59, and 60-86 years) according to the trajectory of handgrip strength changes across the lifespan. Anthropometric, lifestyle, and demographic data are presented as the means with standard deviations (SDs) for continuous variables and percentages for categorical variables. Differences in anthropometric measurements, handgrip strength, lifestyle behaviors, and lung function according to sex were compared using the t-test or the chi-square test. Pearson’s correlation was used to determine the strength of the relationship between handgrip strength and FEV1, FVC, and FEV1/FVC among different sex and age groups. Multiple regression analysis was used to determine the effect of handgrip strength on FEV1, FVC, FEV1/FVC, FEV1% predicted, FVC% predicted, and FEV1/FVC% predicted in different age groups. The models were adjusted for age, height (only for FEV1, FVC, and FEV1/FVC), weight, and smoking status. Multicollinearity was assessed using variance inflation factors. To further investigate the predictive value of handgrip strength on age for abnormal lung function, the participants were then categorized into two age groups (25-59 years and 60-86 years). Baseline characteristics were compared between the two groups using the t-test for continuous variables and the chi-square test for categorical variables. Univariate and multivariate binary logistic regression analyses were performed to analyze the odds ratios (OR) and 95% confidence intervals (95% CI) per 5 kg of handgrip strength for abnormal lung function, stratified by sex and age. Model 1 was adjusted for weight. Model 2 was additionally adjusted for hypertension. Model 3 was further adjusted for lifestyle behaviors (smoking, regular exercise, and alcohol intake). Statistical significance was set at p < 0.05. Statistical analyses were performed using IBM SPSS Statistics, version 29.0 software (IBM Corp., Armonk, NY, USA).

Ethics approval and consent to participate

This study was approved by the Research Ethics Committee of the Faculty of Medicine at the University of Tsukuba (approval number: 1000). The study was conducted in accordance with the Declaration of Helsinki, and subjects provided written informed consent to participate.

## Results

**Figure 1:**
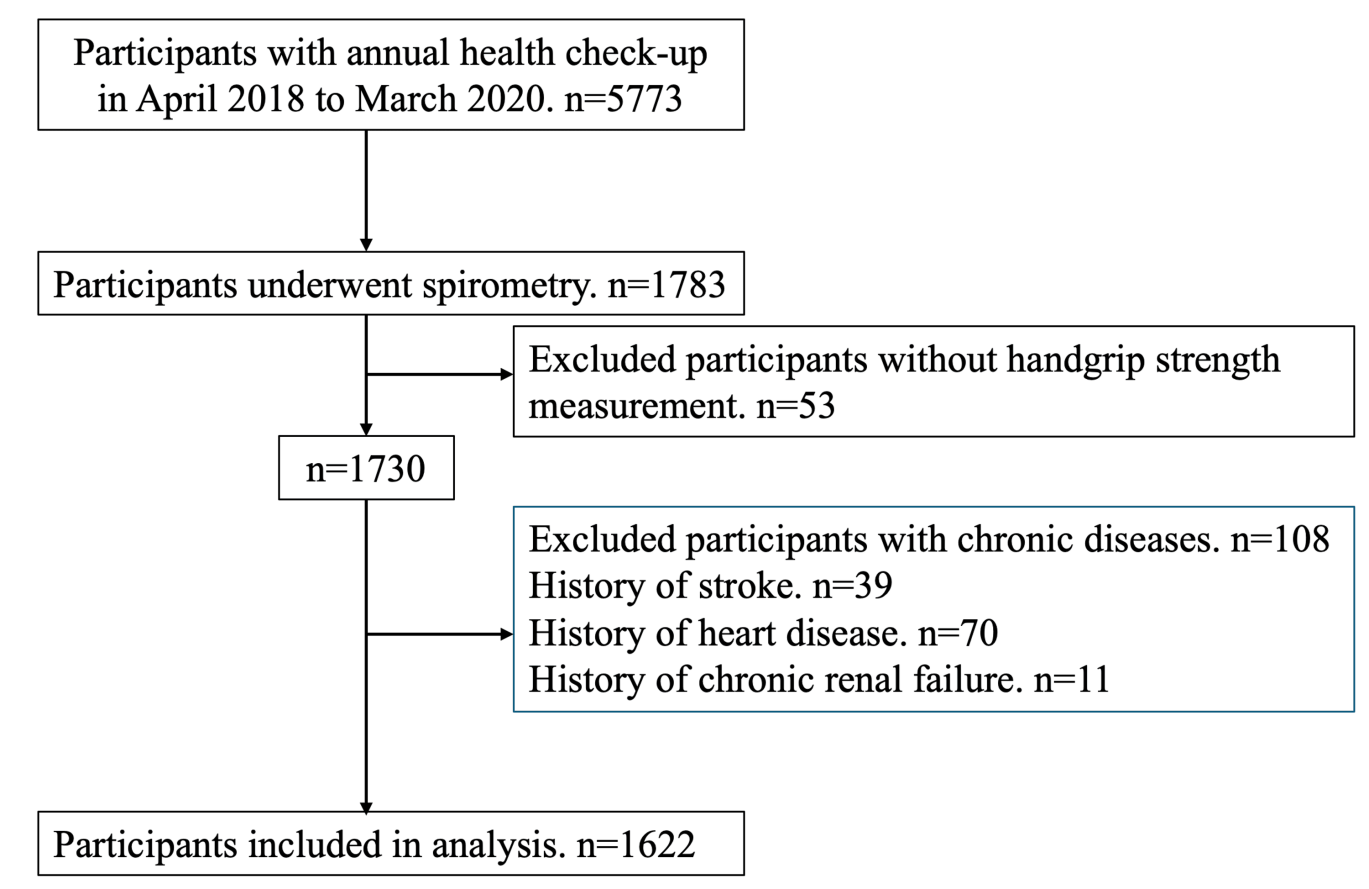
Study flowchart.

A total of 5773 individuals underwent health checkups in the aforementioned setting between April 2018 and March 2020. Among them, only 1783 individuals underwent spirometry. The study excluded individuals who did not undergo measurement of handgrip strength (n = 53), as well as those who had a history of stroke (n = 39), heart disease (n = 70), or chronic renal failure (n = 11). Some individuals had more than one chronic disease; therefore, the total number of excluded participants did not equal the sum of those excluded for each condition. Finally, 1622 individuals were included in the study. A flowchart of the study is shown in Figure 1.

**Figure 2:**
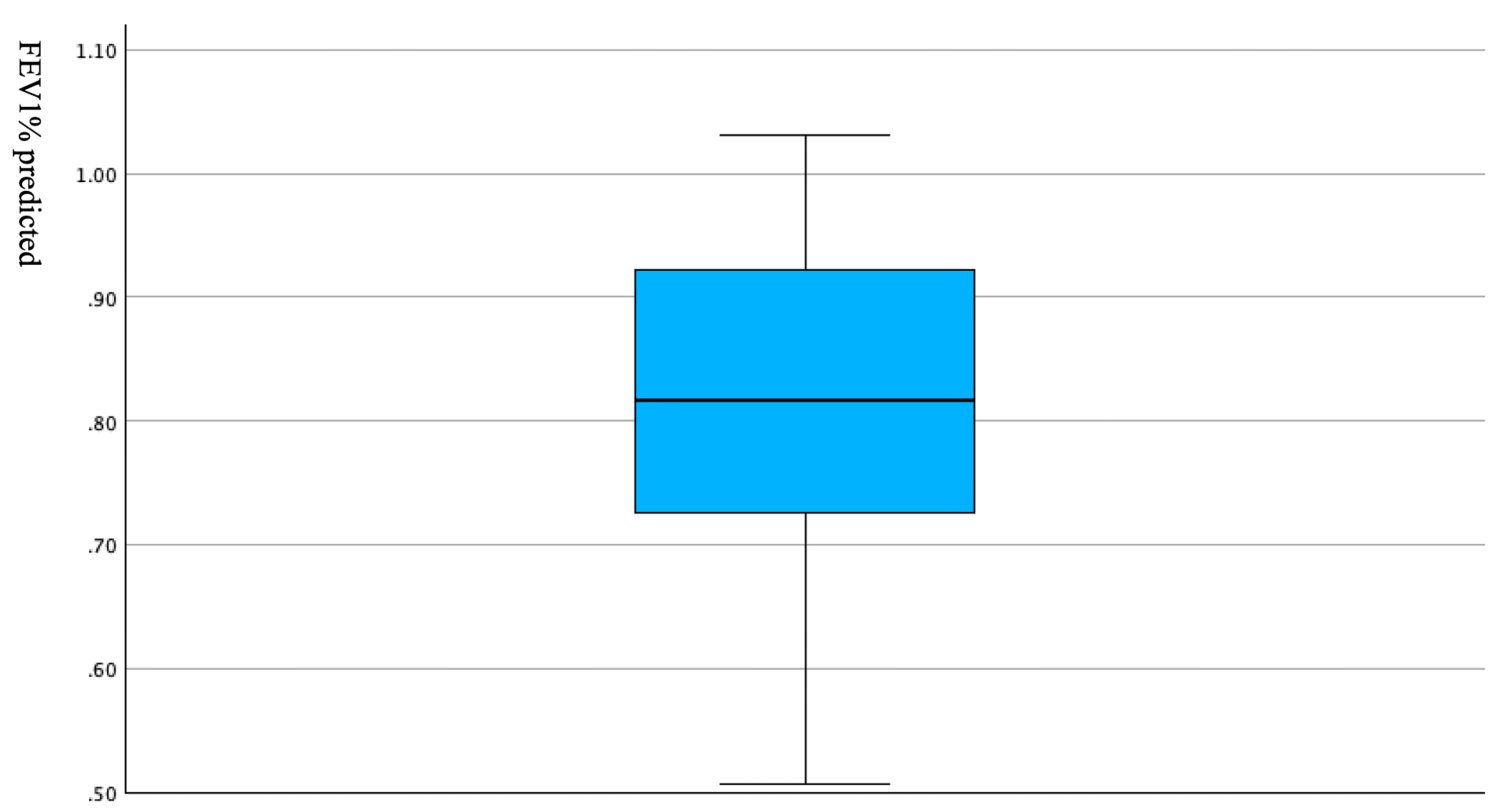
Box plot of FEV1% predicted among suspected COPD patients. FEV1%: forced expiratory volume in 1 second, percent predicted; COPD: chronic obstructive pulmonary disease

To explore the potential impact of undiagnosed COPD on the study results, the FEV1% predicted distribution in smokers aged > 40 years with FEV1/FVC < 70% was plotted. As shown in Figure 2, the median value of FEV1% predicted in this subgroup was approximately 82%, with the lower extreme values still exceeding 50% (mild or moderate severity). Severe cases, which typically present with an FEV1% predicted < 50%, were not observed. This subgroup accounted for 2.15% of the overall study population.

Table 1 shows the characteristics of the study participants. A total of 996 participants (61.4%) were men, with a mean age of 55.1 years (SD 11.7 years). The men were slightly younger than the women (54.6 years vs. 56.0 years; p = 0.018). The proportion of middle-aged and older participants was greater than that of younger participants. Over 60% of the participants had a normal BMI; the prevalence of being overweight was greater among men than women, whereas the proportion of underweight individuals was lower among men. Among the participants, 37.7% had hypertension, 5.0% used antidiabetic agents, and 16.0% used anticholestatic agents. No significant differences were observed in the prevalence of these underlying conditions between men and women. More men reported smoking and alcohol intake, whereas the proportion of men and women with regular exercise habits was similar.

**Table 1:** Characteristics of study participants. *p < 0.05; **p < 0.01. Continuous variables are presented as the means ± SDs, categorical variables are presented as numbers (%). Missing: *1 (n = 1); *2 (n = 3); *3 (n = 5)

Variable	Total (n = 1622)	Men (n = 996)	Women (n = 626)	Test
Age (years)	55.1 ± 11.7	54.6 ± 11.7	56.0 ± 11.7	t = -2.37; p = 0.018*
Height (cm)	164.4 ± 8.9	169.5 ± 6.1	156.3 ± 6.1	t = 42.58; p < 0.001**
Weight (kg)	64.7 ± 12.6	70.2 ± 10.8	56.0 ± 9.9	t = 26.70; p < 0.001**
BMI (kg/m^2^)	23.8 ± 3.5	24.4 ± 3.3	22.9 ± 3.7	t = 8.28; p < 0.001**
BMI category				
Underweight	80 (4.9%)	17 (1.7%)	63 (10.1%)	χ² = 78.85; p < 0.001**
Normal	987 (60.8%)	584 (58.6%)	403 (64.4%)	
Overweight	555 (34.3%)	395 (39.7%)	160 (25.6%)	
Handgrip strength (kg)	34.1 ± 9.5	40.2 ± 6.1	24.4 ± 4.4	t = 60.59; p < 0.001**
Hypertension	612 (37.7%)	367 (36.8%)	245 (39.1%)	χ² = 0.86; p = 0.354
Use of antidiabetic agents^*1^	81 (5.0%)	52 (5.2%)	29 (4.6%)	χ² = 0.29; p = 0.593
Use of anticholestatic agents	259 (16.0%)	153 (15.4%)	106 (16.9%)	χ² = 0.71; p = 0.400
Current smoking	269 (16.6%)	195 (19.6%)	74 (11.8%)	χ² = 16.72; p < 0.001**
Regular exercise^*2^	510 (31.5%)	298 (29.9%)	212 (34.0%)	χ² = 3.00; p = 0.083
Alcohol intake^*3^				
Daily	402 (24.9%)	286 (28.8%)	116 (18.6%)	χ² = 55.63; p < 0.001**
Occasional and rarely	1215 (75.1%)	708 (71.2%)	507 (81.4%)	

Table 2 presents the baseline characteristics of the study population stratified by age groups (< 60 years and ≥ 60 years). Among the participants, 979 individuals (60.4%) were younger than 60 years. There were no significant differences in sex distribution, BMI, or smoking status between the two groups. However, compared with participants aged ≥ 60 years, those < 60 years of age exhibited significantly greater height, weight, and handgrip strength, as well as a lower prevalence of underlying diseases.

**Table 2:** Characteristic of study participants by age groups. *p < 0.05; **p < 0.01. Continuous variables are presented as the means ± SDs, categorical variables are presented as numbers (%). Missing: *1 (n = 1); *2 (n = 3); *3 (n = 5)

Variable	25-59 years (n = 979)	60-86 years (n = 643)	Test
Men	607 (62.0%)	389 (60.5%)	χ² = 0.37; p = 0.543
Height (cm)	166.2 ± 8.5	161.6 ± 8.7	t = 10.54; p < 0.001**
Weight (kg)	66.4 ± 13.1	62.1 ± 11.1	t = 6.96; p < 0.001**
BMI (kg/m^2^)	23.9 ± 3.7	23.7 ± 3.2	t = 1.30; p = 0.193
BMI category			
Underweight	51 (5.2%)	29 (4.5%)	χ² = 2.10; p = 0.351
Normal	582 (59.4%)	405 (63.0%)	
Overweight	346 (35.3%)	209 (32.5%)	
Handgrip strength (kg)	35.3 ± 9.5	32.3 ± 9.1	t = 6.17; p < 0.001**
Hypertension	315 (32.2%)	297 (46.2%)	χ² = 32.44; p < 0.001**
Use of antidiabetic agents^*1^	41 (4.2%)	40 (6.2%)	χ² = 3.41; p = 0.065
Use of anticholestatic agents	119 (12.2%)	140 (21.8%)	χ² = 26.75; p < 0.001**
Current smoking	171 (17.5%)	98 (15.2%)	χ² = 1.39; p = 0.238
Regular exercise^*2^	269 (27.5%)	241 (37.7%)	χ² = 18.58; p < 0.001**
Alcohol intake^*3^			
Daily	217 (22.3%)	185 (28.8%)	χ² = 9.21; p = 0.010*
Occasional and rarely	758 (77.7%)	457 (71.2%)	

Table 3 presents the spirometry findings of the participants. As expected, men had higher FEV1 and FVC values than women; however, no differences were observed in the percentage of predicted values between the sexes. In terms of the FEV1/FVC ratio, a common indicator of airway obstruction, men had significantly lower FEV1/FVC and FEV1/FVC% predicted than women. When the LLN was used to classify abnormal lung function, a smaller proportion of men had abnormal FEV1/FVC and total lung function than women (13.9% vs. 17.6%, p = 0.043).

**Table 3:** Spirometry parameters and lower limit of normal (LLN). *p < 0.05; **p < 0.01. Continuous variables are presented as the means ± SDs, and categorical variables are presented as numbers (%). FEV1: forced expiratory volume in 1 second; FVC: forced vital capacity; L: liter; % predicted: percent predicted

Variable	Total (n = 1622)	Men (n = 996)	Women (n = 626)	Test
FEV1				
L	2.90 ± 0.75	3.27 ± 0.66	2.32 ± 0.46	t = 34.32; p < 0.001**
% predicted	101.09 ± 13.67	100.89 ± 13.93	101.41 ± 13.23	t = -0.75; p = 0.452
FVC				
L	3.66 ± 0.89	4.15 ± 0.71	2.89 ± 0.51	t = 41.90; p < 0.001**
% predicted	102.07 ± 12.54	102.22 ± 12.54	101.82 ± 12.56	t = 0.63; p = 0.529
FEV1/FVC				
%	79.23 ± 6.81	78.55 ± 6.98	80.30 ± 6.39	t = -5.10; p < 0.001**
% predicted	99.20 ± 8.74	98.49 ± 8.08	100.34 ± 9.59	t = -4.02; p < 0.001**
LLN (FEV1)	96 (5.9%)	67 (6.7%)	29 (4.6%)	χ² = 3.03; p = 0.082
LLN (FVC)	68 (4.2%)	44 (4.4%)	24 (3.8%)	χ² = 0.33; p = 0.568
LLN (FEV1/FVC)	169 (10.4%)	89 (8.9%)	80 (12.8%)	χ² = 6.08; p = 0.014*
LLN (Total)	248 (15.3%)	138 (13.9%)	110 (17.6%)	χ² = 4.10; p = 0.043*

Across the three age groups, the correlation coefficients between FEV1, FVC, and handgrip strength were approximately 0.7, indicating a strong association. After stratification by sex, the correlation coefficients decreased to approximately 0.3-0.45. The correlation coefficients between FEV1/FVC and handgrip strength were negative across all three age groups; however, none of these correlations reached statistical significance except in women aged 25-44 years.

The results of the multiple linear regression analysis are presented in Table 4. After adjusting for age, height (only for FEV1, FVC, and FEV1/FVC), weight, and smoking status, handgrip strength was significantly positively associated with FEV1, FEV1% predicted, FVC, and FVC% predicted across all age groups. Additionally, handgrip strength was significantly positively associated with FEV1/FVC% predicted in the 60-86 years age group and significantly negatively associated with that in the 45-59 years age group.

**Table 4:** Multiple linear regression models for lung function and handgrip strength. *p < 0.05; **p < 0.01. Values are presented as β-coefficients with a 95% confidence interval (CI). Adjusted for weight and smoking status; additionally adjusted for age and height in FEV1, FVC, and FEV1/FVC. FEV1: forced expiratory volume in 1 second; FVC: forced vital capacity; % predicted: percent predicted

Variable	25-44 years	45-59 years	60-86 years
Total			
FEV1	0.023 (0.015-0.030)**	0.026 (0.021-0.031)**	0.019 (0.014-0.024)**
FEV1% predicted	0.003 (0.001-0.004)*	0.001 (0.000-0.003)*	0.002 (0.000-0.03)*
FVC	0.031 (0.023-0.038)**	0.033 (0.026-0.039)**	0.026 (0.020-0.032)**
FVC% predicted	0.004 (0.001-0.004)*	0.002 (0.001-0.004)**	0.002 (0.001-0.004)**
FEV1/FVC	-0.076 (-0.166-0.014)	-0.006 (-0.085-0.072)	-0.040 (-0.131-0.051)
FEV1/FVC% predicted	-0.080 (-0.174-0.013)	-0.298 (-0.382 - -0.214)**	0.151 (0.055-0.246)*
Men			
FEV1	0.010 (0.000-0.021)*	0.012 (0.004-0.020)*	0.006 (-0.002-0.015)
FEV1% predicted	0.003 (0.000-0.006)*	0.004 (0.001-0.006)*	0.003 (0.000-0.006)*
FVC	0.017 (0.006-0.029)*	0.011 (0.001-0.021)*	0.006 (-0.004-0.015)
FVC% predicted	0.003 (0.00-0.006)*	0.003 (0.000-0.005)*	0.002 (-0.000-0.004)
FEV1/FVC	-0.073 (-0.195-0.050)	0.082 (-0.038-0.202)	0.046 (-0.088-0.179)
FEV1/FVC% predicted	-0.063 (-0.133-0.006)	0.057 (-0.088-0.202)	0.107 (-0.052-0.266)
Women			
FEV1	0.016 (0.001-0.031)*	0.015 (0.004-0.025)*	0.020 (0.011-0.028)**
FEV1% predicted	0.006 (0.001-0.011)*	0.006 (0.002-0.010)*	0.008 (0.004-0.012)**
FVC	0.030 (0.014-0.046)**	0.021 (0.009-0.032)**	0.028 (0.018-0.039)**
FVC% predicted	0.008 (0.004-0.013)**	0.006 (0.003-0.010)**	0.010 (0.006-0.013)**
FEV1/FVC	-0.278 (-0.532-0.023)	-0.077 (-0.267-0.114)	-0.114 (-0.309-0.081)
FEV1/FVC% predicted	-0.119 (-0.471-0.233)	-0.075 (-0.318-0.168)	0.166 (-0.094-0.426)

After sex stratification, handgrip strength was positively associated with FEV1, FEV1% predicted, FVC, and FVC% predicted across all age groups in women. Among men, handgrip strength was positively associated with FEV1, FEV1% predicted, FVC, and FVC% predicted in the 25-44 and 45-59 years age groups. However, in men aged > 60 years, a significant positive association was observed only with the FEV1% predicted. The associations between handgrip strength and FEV1/FVC and FEV1/FVC% predicted were similar to the results of the correlation analysis, showing a negative relationship among women and men aged 25-44 years. In contrast, a positive association was observed in men aged 45-59 and 60-86 years; however, these differences were not statistically significant.

Logistic regression analyses of the association between handgrip strength and abnormal lung function are shown in Table 5. In the sex-stratified analysis after adjusting for weight, hypertension, smoking, regular exercise, and alcohol intake, handgrip strength per 5 kg increase was significantly associated with abnormal lung function in both men and women (men: OR = 0.790, 95% CI = 0.673-0.927, p < 0.01; women: OR = 0.598, 95% CI = 0.461-0.775, p < 0.01). Tables 6-7 present the results of the logistic regression analyses for handgrip strength and abnormal lung function in different age groups for both men and women. When further stratified into younger (< 60 years) and older (≥ 60 years) age groups, for men, the association was not significant in any of the models for the younger group. In the older group, the crude model showed significant associations; however, after adjusting for weight, hypertension, and lifestyle factors, the p-value was borderline. For women aged < 60 years, the significant association between handgrip strength and abnormal lung function remained consistent across all models (OR = 0.621, 95% CI = 0.404-0.955; p < 0.05). However, in women aged > 60 years, the significant relationship between handgrip strength and abnormal lung function became nonsignificant after adjusting for weight and hypertension. After further adjustment for lifestyle factors, the association became significant (OR = 0.673, 95% CI = 0.470-0.964, p < 0.05).

**Table 5:** Logistic regression analysis for abnormal lung function and handgrip strength stratified by sex. **p < 0.01. Values are presented as odds ratio (OR) with 95% confidence interval (CI). Abnormal lung function is defined using the LLN for FEV1, FVC, or FEV1/FVC ratio. FEV1: forced expiratory volume in 1 second; FVC: forced vital capacity; LLN: lower limit of normal

Sex	Crude	Model 1 (weight)	Model 2 (Model 1 and hypertension)	Model 3 (Model 2 and lifestyle factors)
Men	0.768 (0.662-0.891)**	0.795 (0.678-0.932)**	0.785 (0.670-0.920)**	0.790 (0.673-0.927)**
Women	0.593 (0.464-0.758)**	0.614 (0.475-0.793)**	0.615 (0.476-0.795)**	0.598 (0.461-0.775)**

**Table 6:** Logistic regression analysis for abnormal lung function and handgrip strength in men stratified by age groups. *p < 0.05 Values are presented as odds ratio (OR) with 95% confidence interval (CI). Abnormal lung function is defined using the LLN for FEV1, FVC, or FEV1/FVC ratio. FEV1: forced expiratory volume in 1 second; FVC: forced vital capacity; LLN: lower limit of normal

Age group	Crude	Model 1 (weight)	Model 2 (Model 1 and hypertension)	Model 3 (Model 2 and lifestyle factors)
25-59 years	0.866 (0.691-1.086)	0.863 (0.683-1.091)	0.863 (0.682-1.092)	0.865 (0.683-1.097)
60-86 years	0.787 (0.639-0.970)*	0.820 (0.655-1.027)	0.822 (0.655-1.030)	0.809 (0.644-1.016)

**Table 7:** Logistic regression analysis for abnormal lung function and handgrip strength in women stratified by age groups. *p < 0.05 Values are presented as odds ratio (OR) with 95% confidence interval (CI). Abnormal lung function is defined using the LLN for FEV1, FVC, or FEV1/FVC ratio. FEV1: forced expiratory volume in 1 second; FVC: forced vital capacity; LLN: lower limit of normal

Age group	Crude	Model 1 (weight)	Model 2 (Model 1 and hypertension)	Model 3 (Model 2 and lifestyle factors)
25-59 years	0.641 (0.425-0.967)*	0.640 (0.420-0.976)*	0.635 (0.415-0.972)*	0.621 (0.404-0.955)*
60-86 years	0.693 (0.502-0.958)*	0.721 (0.513-1.014)	0.721 (0.514-1.013)	0.673 (0.470-0.964)*

## Discussion

This study revealed that handgrip strength was positively associated with FEV1, FVC, FEV1% predicted, and FVC% predicted among women aged 25-44, 45-59, and 60-86 years in a healthy adult population. Additionally, an increase of 5 kg in handgrip strength was significantly associated with a 37.9% and 32.7% reduction in the ORs for lung function abnormalities in the young and old age groups, respectively. Among men, handgrip strength was positively associated with FEV1, FVC, FEV1% predicted, and FVC% predicted in the 25-44 and 45-59 year age groups; however, no significant association was observed in those aged > 60 years. Nevertheless, the association between abnormal lung function and a 5 kg increase in handgrip strength was not statistically significant in men after age stratification.

The incidence of pulmonary diseases has increased annually, and there has been increasing research on FEV1, FVC, and FEV1/FVC as indicators of lung function, as well as handgrip strength, a simple and reliable screening tool. Handgrip strength and lung function are influenced by common physiological and environmental factors, such as physical activity, nutritional status, and exposure to environmental pollutants. These factors affect muscle mass, respiratory muscle strength, and overall metabolic health, which may contribute to the observed association between muscle strength and lung function. Previous studies have reported an association between handgrip strength and FEV1 and FVC. However, no studies have examined the relationship between handgrip strength and lung function across different sexes and age groups in healthy populations. Studies on Nigerian students aged 16-30 years [21] and Japanese students aged 22-24 years [14] reported a significant association between handgrip strength and FEV1 and FVC, indicating a relationship between handgrip strength and lung function. In the general population, studies from China, Malaysia, South Korea, and Japan have confirmed this association across a wider age range [7-9,22]. In a study on the South Korean population [7], handgrip strength was positively associated with FEV1% predicted and FVC% predicted, suggesting that handgrip strength not only reflects absolute lung function but also relative functional reserve, which is critical for assessing pulmonary health. The findings of the present study are consistent with those of previous studies. However, among elderly individuals aged > 60 years, the findings are inconsistent. For example, a study of hospitalized individuals aged > 70 years in the UK reported a significant association between handgrip strength and FEV1 and FVC in men but not in women [23]. A study of men aged > 60 years living in nursing homes in Turkey revealed no significant association between handgrip strength and FVC% predicted, FEV1% predicted, or FEV1/FVC [24]. In a study of Korean women aged > 65 years, a dose-response relationship was observed between handgrip strength and FEV1 and FVC [10]. This association was observed in women aged > 60 years but not in men aged > 60 years. This discrepancy may largely be due to differences in the health status of the study population. Prior studies of older adults have mostly focused on those with underlying health conditions that may affect the relationship between muscle strength and lung function. A recent review on the relationship between handgrip strength and lung function has shown inconsistent conclusions in unhealthy populations [25]. However, they also found significant associations between lung function and handgrip strength in healthy elderly women, which was corroborated by the results of this study.

In this study, handgrip strength was negatively correlated with FEV1/FVC in men aged 25-44 years and in women across all age groups. Similar results were observed in multiple linear regression analyses, with negative associations observed even with FEV1/FVC% predicted. However, these differences were not statistically significant. The FEV1/FVC ratio is an indicator of airflow obstruction, and the results of the present study do not imply that greater muscle strength leads to more severe airflow obstruction. The reason for this result might be that the association between handgrip strength and FVC was more significant than that with FEV1 [10,26], leading to a decrease in the FEV1/FVC ratio with increased handgrip strength. FVC performance is influenced primarily by inspiratory and expiratory muscle strength [27], which are dominated by the diaphragm and intercostal muscles, which are skeletal muscles [28]. Handgrip strength is positively correlated with respiratory muscle strength [29]. Therefore, individuals with greater handgrip strength tend to have a larger FVC. This finding suggests that while handgrip strength is a reliable indicator of lung function with FEV1 and FVC, it may not be as reliable when assessing airflow obstruction using the FEV1/FVC ratio. A previous study in a Japanese population reported no significant association between handgrip strength and FEV1/FVC [9]. Our study further confirmed that handgrip strength was not associated with FEV1/FVC% predicted across different sexes and age groups.

This study demonstrates an association between handgrip strength and abnormal lung function in women. Diagnosing lung function abnormalities using LLN can avoid underdiagnosis in younger populations and overdiagnosis in older populations and can eliminate sex differences that arise from the use of fixed values for diagnosis [16,30]. The LMS (Lambda-Mu-Sigma) formula, which accounts for distributional characteristics by modeling the skewness (L), mean (M), and coefficient of variation (S), considers the differences in sex and height, thereby reflecting the physiological differences between men and women [19]. In women, their smaller body size leads to lower baseline lung function [12], which may be more sensitive to variations in handgrip strength. In this study population, a greater proportion of men were smokers and obese, which can lead to a decline in lung function and may have a greater impact on lung function than on handgrip strength [31,32]. Additionally, in elderly men and women, after controlling for lifestyle habits in Model 3, the association between handgrip strength and abnormal lung function became stronger than that in Models 2 and 1, which indicates the importance of maintaining good lifestyle habits to preserve lung function. Lung function begins to decline in early adulthood. Low baseline lung function in adulthood and rapid early decline in lung function are risk factors for pulmonary diseases [13]. Although spirometry can be used to diagnose and monitor lung health, it is not routinely performed in early adulthood, and the opportunity for early detection and intervention may be missed. Therefore, incorporating regular handgrip strength assessments into routine health check-ups can serve as an effective screening tool. By establishing a baseline value and tracking changes in handgrip strength over time, healthcare providers can gain valuable insights into the lung health status of individuals in early adulthood and identify those who may require further evaluation or intervention.

The strengths of this study include the recruitment of a relatively healthy population with a broad age range by excluding participants with underlying severe chronic diseases. Additionally, this study used standardized formulas for the Japanese population to calculate the predicted values and determine abnormal lung function, making the findings more generalizable to the Japanese population.

However, this study has several limitations. First, as this was a cross-sectional study, causal relationships could not be established. A longitudinal approach would have been more suitable for examining the temporal relationships and potential causal links; however, the follow-up data collection for lung function testing among this population was significantly disrupted due to the COVID-19 pandemic. Future longitudinal studies are warranted to understand the causal relationship between handgrip strength and lung function. Despite these challenges, cross-sectional data from a health check-up population provide valuable insights into the association between handgrip strength and lung function in a relatively healthy population. Second, the study population consists of individuals participating in health check-ups, which may indicate a higher level of health consciousness compared to the general population. Third, to obtain a larger sample size, this study did not exclude individuals with chronic conditions such as hypertension or smoking. The proportion of these factors in the study population may have affected the results, even though the models were adjusted for these factors. Fourth, because the LLN diagnostic method identified the lowest 5% of the population, the relationship between different types of FEV1, FVC, and FEV1/FVC lung function abnormalities and handgrip strength across different ages and sexes could not be further distinguished, owing to the limited number of cases. Large-scale longitudinal studies are required to verify these findings. Fifth, we were unable to explicitly exclude participants with undiagnosed COPD, as the questionnaire did not include questions regarding COPD history. However, additional analysis focused on smokers aged > 40 years with FEV1/FVC < 70%, and no severe cases were observed. Moreover, the inclusion criteria requiring participation in comprehensive health check-ups likely minimized the impact of severe COPD cases in the study population.

## Conclusions

In conclusion, this study demonstrates a significant positive association between handgrip strength and both the absolute and percentage values of predicted FEV1 and FVC in women across all age groups and in men aged < 60 years. Handgrip strength per 5 kg increase was significantly associated with lower abnormal lung function in women. Regular handgrip strength measurements to track changes in lung function may help detect abnormalities in lung function during early adulthood. Future longitudinal studies are needed to confirm the temporal relationship and assess the clinical utility of handgrip strength in lung function monitoring and early detection of abnormalities.

## Appendices

Appendix A

**Table 8:** Standard questionnaire.

Question	Answer
1. Are you currently taking medication to lower your blood pressure?	1. Yes 2. No
2. Are you currently using insulin injections or medication to lower blood sugar levels?	1. Yes 2. No
3. Are you currently taking medication to lower cholesterol or triglycerides?	1. Yes 2. No
4. Have you ever been diagnosed with stroke (e.g., cerebral hemorrhage, cerebral infarction) or received treatment for it?	1. Yes 2. No
5. Have you ever been diagnosed with heart disease (e.g., angina, myocardial infarction) or received treatment for it?	1. Yes 2. No
6. Have you ever been diagnosed with chronic kidney disease or kidney failure, or are you currently undergoing dialysis?	1. Yes 2. No
7. Do you currently smoke regularly?	1. Yes 2. No
8. Have you engaged in moderate exercise for at least 30 minutes, two or more times a week, for over a year?	1. Yes 2. No
9. How often do you consume alcoholic beverages (e.g., sake, shochu, beer, whisky)?	1. Daily 2. Occasionally 3. Rarely
